# Optogenetics: Emerging strategies for neuropathic pain treatment

**DOI:** 10.3389/fneur.2022.982223

**Published:** 2022-12-01

**Authors:** Siyu Li, Xiaoli Feng, Hui Bian

**Affiliations:** ^1^Department of Physiology, Faculty of Basic Medical Science, Kunming Medical University, Kunming, Yunnan, China; ^2^Key Laboratory of Animal Models and Human Disease Mechanisms of the Chinese Academy of Sciences and Yunnan Province, Kunming Institute of Zoology, Chinese Academy of Sciences, Kunming, Yunnan, China

**Keywords:** neuropathic pain, optogenetics, treatment, neuromechanism, potential

## Abstract

Neuropathic pain (NP) is a chronic health condition that presents a significant burden on patients, society, and even healthcare systems. However, in recent years, an emerging field in the treatment of neuropathic pain – optogenetic technology has dawned, heralding a new era in the field of medicine, and which has brought with it unlimited possibilities for studying the mechanism of NP and the treatment of research. Optogenetics is a new and growing field that uses the combination of light and molecular genetics for the first time ever. This rare combination is used to control the activity of living cells by expressing photosensitive proteins to visualize signaling events and manipulate cell activity. The treatments for NP are limited and have hardly achieved the desirable efficacy. NP differs from other types of pain, such as nociceptive pain, in that the treatments for NP are far more complex and highly challenging for clinical practice. This review presents the background of optogenetics, current applications in various fields, and the findings of optogenetics in NP. It also elaborates on the basic concepts of neuropathy, therapeutic applications, and the potential of optogenetics from the bench to the bedside in the near future.

## Introduction

Neuropathic pain (NP) is defined by the International Association for the Study of Pain (IASP) as the “pain caused by a lesion or disease of the somatosensory nervous system” (1, 2). The definition of NP is very broad based owing to the complexities it presents and should also include in its fold several characteristics and traits of NP (3). However, many definitions put forth to describe NP are still inadequate and even missing appropriate words. NP is caused by neuronal lesions, and treatment methods to bring about an effective cure for this disease are very limited. The prevalence of chronic pain in patients with neuropathological characteristics, according to large-scale epidemiological findings, is ~7–10% (4). In recent years, as the growing trend of the global population is toward aging, and as their survival rate has prolonged after the treatment of various diseases, the prevalence of NP too has increased further. Studies have shown that patients with NP often experience anxiety, depression, sleep disorders, and other symptoms (5). These debilitating symptoms seriously affect their quality of life when compared to patients who experience other types of chronic pain, such as: inflammatory pain, fibromyalgia, non-specific low back pain, etc. NP has numerous causes and complex neuromechanisms, so far, that these neuromechanisms of NP have not been fully understood and even remain to be elucidated. This review describes a few common neuromechanism views of NP.

Furthermore, the treatment of NP that is being given to these NP patients still presents a great challenge for clinical practice, as the clinicians have to face a plethora of problems while engaging and handling such patients. Pharmacotherapy is the main and important treatment method to bring about a drastic cure in these patients, but the current treatment only produces limited remission (30–50%) that too only for a portion of patients (6). Pharmacotherapy also brings along with it the problem of drug dose and side effects which could also affect these patients. Non-pharmacological treatments have small side effects, but some treatments lack clinically reliable evidences, while in some cases, the combination of multiple drugs or treatments can achieve better results. In recent years, an emerging field of neurogenic pain treatment – optogenetics is the newest and most recent treatment method for the treatment of NP and has started making its imprint in treating NP patients, and there is so much unlimited untapped potential for the treatment of NP it offers to treat patients. Thanks to the development of photogenetics, researchers can now control the treatment-related activities in defined neuronal populations and projections, while examining their effects on behavior and physiology. Unlike pharmacology and disease-based intervention, optogenetics also opens up causal investigation and specificity for the rapid time scale of natural nervous system communication (7, 8).

The results of many research works corroborate the role of optogenetics and have also put forth a certain affirmation of the role of optogenetics. In this review, we provide an overview of the basic concepts of optogenetics and the active application of optogenetic techniques in the treatment of NP in recent years.

### Neuromechanism for NP

Neuropathic pain (NP), which is triggered by the impaired somatosensory system, attributes its origin to various health-related causes. These causes are diverse, arising from a wide gamut of diseases, such as diabetes, metabolic diseases, tumors, spinal cord injury, etc. After a nerve injury takes place, the nerve fibers change at multiple levels, resulting in disturbances during transmission to the brain and spinal cord, and involves issues such as altered pain thresholds and signal effects (9). There are several points regarding the pathogenesis of NP that merit our attention and need to be studied in depth.

### Neuronal activity

#### Abnormalities in neuronal activity

Changes in nerve fiber secretions are closely associated with pain. As regards to neurons, a popular view was held to express the fact that nociceptive afferents inhibit the flow of harmful information from the spinal cord into the brain by activating spinal inhibitory neurons and that the imbalance between excitatory and inhibitory neurotransmission serves as the source of ectopic pain (10). The hypothesis has been confirmed by many studies in recent decades, with increasing evidence suggesting that the balance between excitation and inhibition in spinal circuits is disrupted in NP (11). This reasoning was corroborated further by experimental studies, which were conducted to prove that the transplantation of GABAergic precursor cells into the dorsal spinal horn reduced neurological mechanical pain (12). At present, the research dynamics of the mechanism of neurons in neuronal circuits are relatively active, and the results of the research works are relatively rich in values and data. However, it has not been explained accurately in which neuronal circuits do the neurons participate.

### Ion channels

Transient receptor potential (TRP) family of ion channels and other ion channels, such as acid-sensing ion channels adenosine triphosphate (ATP)-gated purine channel [ATP-PRX (peroxiredoxin)], has highly specific involvement in various types of nociceptive stimulations. At this time, different types of sodium channels play a significant role, by magnifying the point of the receptor, so as to trigger the depolarization of the action potential. Notably, these ion channels are all not only strongly regulated by post-translational modifications and transcriptional levels, but can also be deregulated upon nerve damage. However, the present study does not prove the main connection of TRP channels with NP syndrome, but pharmacologically related studies prove that blocking TRP channels in rodent models relieves neuropathic allergy (13). Studies have shown that knockout mouse sodium channels in root ganglion neurons effectively slow down pain (14, 15). This suggests that there is a connection between NP and ion channels and that NP is strongly associated with ion channels. For instance, several studies have shown that downregulation of potassium channels was observed in the experimental animal models of NP (16). Overexpression or downregulation of sodium channels is closely related to threshold and ectopic activity, which also provides a huge therapeutic space for subsequent intervention of sodium channel blockers.

### Immune cells

Here, we focus on some of the most recent studies related to NP. T cells in immune cells have a very important role to play, and studies have shown that mice lacking T cells completely lack the ability to produce neuropathic abnormal pain after a nerve injury (17). Furthermore, other studies have demonstrated that angiotensin 2 mediates the attenuation of neurological ectopic pain in the expression of invading macrophages at the site of nerve injury (18). These studies suggest a great therapeutic potential of immune cells in targeting NP and warrant further exploration.

### Mitochondrial factors

Notably, the dysfunction of mitochondrial function in peripheral neurons can manifest in various NP types in different animal models (19, 20), and this reasoning reflects the important role of mitochondria in NP. Mitochondria are closely related to the production of reactive oxygen species (ROS), and any mitochondrial dysfunction leads to an energy deficiency, triggering off many potential crises. Mitochondria and reactive oxygen species (ROS) have only recently emerged in the field of NP and have aroused the widespread interest of researchers in their direction. Studies have shown that drugs that cause a nitrite breakdown also serve the function to reduce neuropathic ectopic pain during chemotherapy for cancer patients (21). Passakorn et al. (22) demonstrated an additional benefit by supplementing coenzyme Q10 (CoQ10) on pain relief in patients with pregabalin-treated fibromyalgia, which may be achieved by improving mitochondrial function, reducing inflammation, and reducing brain activity. In conclusion, mitochondria have a potential therapeutic space for the treatment of interventional NP, but their more precise mechanism needs more intensive investigation.

We describe several neural mechanisms associated with NP that are currently well recognized. Actually, the neuromechanisms underlying NP remain unexplored, because of its diverse etiology and numerous systems involved. Moreover, the transmission of pain signals is regulated by complex large neural networks, making it more difficult to carry out research on neural mechanisms. Of course, the views discussed previously also bring a direction toward new therapy in the treatment of NP, because optogenetics can also help with research into NP pathways.

### General treatment of neuropathic pain

At present, the treatment approach of NP is mainly divided into pharmacotherapy and non-pharmacotherapy types, and both the treatment methods have their own inherent advantages and disadvantages. A broad consensus has been reached on the drug treatment to be administered to the patient (23), as this drug treatment is mostly based on the urgency and severity of the patient's condition and the specific course of the disease. Hence, generally drugs are divided into three echelons. However, either tricyclic antidepressants or opioid analgesics, as the first choice of drugs (24), or the third-line drugs, such as cetin, NMDA (N-methyl-D-aspartate) receptor antagonists, and local capsaicin (25, 26), have been greatly restricted in their usage due to the clinical side effects caused by them, and the clinical response is general. With regard to the non-pharmacotherapy type, whether it is traditional acupuncture (27, 28), physical therapy (29, 30), transcutaneous electrical nerve stimulation (31–33) or the virtual reality (VR) (34), spinal cord stimulation (35, 36) and other treatment methods, although its side effects have been greatly reduced (37, 38)and some results have even been achieved by pharmacotherapy, the effect of its clinical treatment has not yet brought the effect of the much-desired ideal treatment to patients. However, the clinical efficacy of some therapeutic methods still needs clarity (39), and even the relevant therapeutic mechanism also needs to be explicit (40). In a word, the therapeutic mechanism and efficacy of these non-pharmacotherapy options in the field of neuropathic pain still need to be corroborated by a large number of basic experiments and clinical experiments.

As we all know, optogenetics has become a new technology in recent years, which is helpful to explore the mechanism of most diseases. We notice that this technology still has huge untapped potential and offers scope for utilizing the unlimited prospects in the application of neurological diseases and other diseases in the future.

### Background in optogenetics

Optogenetics is an emerging field, which boasts of a hitherto unknown, amazing combination of optics and genetics. Optogenetics mainly acts on the corresponding proteins by currents that are generated by transfer to photosensitive proteins (41, 42). Optogenetic activators, such as channelrhodopsin, halorhodopsin (halogenated rhodopsin), and archaerhodopsin (Arch)., are used to control neurons, and the monitoring of neuronal activity is performed through genetically encoded ions (e.g., calcium) or membrane voltage sensors. The effectors in this system are light, with the advantage of working at high spatial and temporal resolution at multiple wavelengths and locations (43, 44). The first step in the development of optogenetic techniques was started way back in 1971 when Oesterhelt and Stoeckenius discovered that the bacterium rhodopsin, a purulentin from the halogen violet membrane of Halogen bacillus, could pump protons under light (45). Later, Sugiyama and Mukohata identified another member of the opsin family in 1984 – halogenated rhodopsin (46), while Nagel et al. identified channelrhodopsin purpurulite in 2002 (47). A major breakthrough was made in the field of optogenetics after the discovery that neurons respond to light when microbial opsin genes were introduced without any other component (48).

Optogenetics includes three main optogenetic tools, including (1) photoactivated proteins; (2) light; (3) delivery mode, virus-mediated gene delivery system is currently one of the most commonly used methods. When applying optogenetic techniques, we benefit from a number of advantages. A significant advantage one can derive from optogenetics is that rapid activation and silencing of expressed proteins can be achieved without the use of chemicals (41, 49). Optogenetic techniques are currently receiving widespread attention from researchers and are actively being applied in various fields. Photogenetics is the result of the fruitful combination of optics and genetic engineering, which maximizes the advantages of each discipline to the fullest extent possible. These advantages are multifold such as optical control by manipulating the wavelength and light intensity on the millisecond time scale, as well as specific gene expression and gene product transport with subcellular accuracy. It is not possible to realize this kind of fine adjustment by traditional methods. Therefore, optogenetics technology has brought about a revolution to neuroscience (50).

### Applications of optogenetics

Optogenetics has been vigorously developing in recent years, such as giving light, facility to be controlled in real time, and becoming closer to the natural environment (51, 52). One branch of medicine that has benefited the most in the field of medicine from optogenetics is ophthalmology. In vision studies, optogenetics makes it possible to impart and infuse light sensitivity to different retinal cell types, thus providing a new perspective on vision restoration in various inherited retinal degenerative diseases. Bi et al. (53) first showed that after complete photoreceptor degeneration, light sensitivity can be restored by the expression of channel rhodopsin-2 (ChR2) in retinal ganglion cells, and a number of other studies have since proven this scientific advancement (54–57). Optogenetics has also been widely used in the treatment of neurological diseases in recent years. Alzheimer's disease (AD) is characterized by the presence of amyloid β (Aβ) plaques and neurofiber tau tangles (58), Lim et al. (59) developed fluorescently labeled optogenetically activated Aβ peptides that can oligomerize *in vitro* during light exposure. Kaur et al. (60) had used a similar method to produce Aβ aggregations *in vivo*. There are also studies that suggest that optogenetic inhibition of pyramidal cells (PCs) in the CA1 region of the dorsal side of the hippocampus (61) or by deinsuppression of somatostatin-positive (SST) cells (62) reversibly disrupts memory acquisition. In the field of neuroscience, scientists have found that optogenetic techniques reduce circuit noise associated with schizophrenia to enhance the performance of cortical circuit (63, 64). Optogenetics shows great potential in various fields, and of course, NP is no exception.

The combination of optogenetics and electrophysiology has also brought about sweeping reforms in the field of neuroscience. Traditional *in vivo* electrophysiology is also difficult to relate to specific cell types defined by genetics or connectivity, so it serves as an important and universal technology integration to combine *in vivo* electrophysiological recording with optogenetics. It is reported that using this method, the activation of the basolateral amygdala (BLA) stimulates the nucleus accumbens (NAc) to drive reward seeking (65). The activation of GABAergic cells from the extended amygdala inhibits the lateral hypothalamic neurons, leading to an increased food consumption. In addition, the medial prefrontal cortex (mPFC) requires ventral hippocampus input to encode the target position (66) and the aversion (open) and safety (closed) spaces (67) in the elevated maze. Similar neurological events can occur across circuits; for instance, the bed nucleus of the stria terminalis (BNST) uses the BLA input to code the enclosed space in the same maze (68). The combination of electrophysiology and photogenetics has unlimited potential in the field of neuroscience. However, neuropathic pain is an extremely complex disease, with complex symptoms and causes. The mechanism and treatment of neuropathic pain still merit a lot of work to be done by researchers to explore their unknown potential.

### Optogenetics in neuropathic pain

Recently, optogenetic combination with NP has also been developing increasingly. When compared to conventional electrical stimulation, optogenetics eliminates the critical step of placing electrodes in the brain with a relatively homogeneous group of neurons (69). But the millisecond-level time accuracy of electrical stimulation displayed by optogenetic techniques stands unmatched. The application of optogenetics in the field of neuropathy has shown us the great potential of utilizing optogenetics in the treatment of NP and the exploration of mechanisms.

In recent years, a large number of studies have combined this technique of optogenetics in the field of NP and have brought to light various novel features and potential of optogenetics in NP treatment by way of new methods for pain relief. For example: in an earlier study, Daou et al. (70) demonstrated the role of optogenetics in suppressing pain using a binary genetic approach, delivering ChR2 channels to peripheral nociceptors in the Nav1.8-Cre transgenic mouse line. Notably, in their experiments, the free-moving small pains were caused to move in a non-invasive and remote manner. In the two other studies, in the NP model, *in vivo* stimulation of the halogenated rhodopsin (eNpHR3.0) channel of the yellow photosensitive third-generation chloride pump successfully prevented pain (71, 72), thus highlighting the therapeutic potential of optogenetics. In studies of neuropathic pain-like behaviors controlled by the parabrachial nucleus circuit, optogenetic activation of glutamatergic or inhibition of γ-aminobutyric acid (GABA)-capable lateral parabrachial nucleus (LPBN) neurons induced neuropathic pain-like behavior in young mice (73). The medial prefrontal cortex (mPFC) is a region of the brain that is involved in the emotional component of pain that undergoes plasticity during the development of chronic pain. Dopamine (DA) is the key neuromodulator in the middle cortical circuit that regulates working memory and loathing. In this study on brain pathways, the results demonstrated that phase activation of DA input from the ventral cover area (VTA) to mPFC reduced mechanical hypersensitivity in NP states (74). The central amygdala (CeA) in the brain is also an important area for mood control, and the results of Hua et al. show that the optogenetic activation of CeA effectively inhibits the reflex and self-healing behaviors caused by pain in sensory patterns and eliminates the mechanical (high) sensitivity induced by NP (75). Similar results were obtained in another study of this region (amygdala), where optogenetic manipulation of adrenocorticotropic hormone-releasing factor (central amygdala corticotropin-releasing factor (CeA-CRF)) neurons in CeA modulates NP and controls pain and anxiety-like behavior in rats (76). Gadotti et al. (77) confirmed that optogenetic techniques could also be operated through modulation of the medial prefrontal cortex function to treat NP. Xiong et al. (78) have found that altering neuronal properties and normalizing their cortical excitability relieves pain. Stimulation of the anterior cingulate cortex enhances excitatory synaptic transmission of the spinal cord and leads to pain hypersensitivity responses. Studies have demonstrated that inhibition of this type by optogenetic methods can produce an anti-injury effect and can also reduce nerve damage caused by synaptic enhancement (79). Recently, there have been many reports of successful control of pain in animal models mediated by optogenetic techniques (80–83). These experimental studies suggest the infinite potential of optogenetics in NP treatment. Optogenetic precision, being controllable, and deliberate selectivity are its advantages (84).

The real challenge of optogenetics is to target the mechanisms of NP, pinpoint its damaged nociceptive neurons, understand nociceptors (as the main subset of units of pain, with receptors and ion channels that can detect stimuli for potential damage) and circuits. Optogenetics involves inducing neuronal expression of light-activated membrane proteins, the activation of which can directly turn on or off neurons by depolarization (e.g., channel rhodopsin-2, ChR2) or hyperpolarization (e.g., haloopeopreum purpurulite), respectively, and opening a cascade of G protein-coupled receptor (GPCR) signals (85). Therefore, it is necessary to understand the genetics of pathological neurons compared with normal functional neural circuits. The complete transcriptome of trigeminal ganglia (TG) and dorsal root ganglia (DRG) in adult mice was analyzed to gain insight into the expression of ion channels and G-protein-coupled receptors (GPCRs) under physiological and pathophysiological conditions. This analysis suggests that given the complex etiologies of ganglion pathological changes, conventional strategies to inhibit individual ion channels or inflammatory processes are less useful (86–88).

All in all, optogenetics presents both an opportunity and a challenge. For example, optogenetics is currently restricted to experiments on animals only, so how can it be extended to experiments conducted on human beings? Optogenetic technology can be effectively implemented in rodents most of the time, but it has been difficult to effectively implement this optogenetic technology in primates for many years now. The reason for this difficulty is attributed to the all-time flexibility of primates, which makes it even more difficult to translate experiments into clinical practice. There are also limitations regarding the way photosensitive proteins are entered and the length of optogenetics to be maintained. Is there a way to make the effect longer or even permanent? There is no denying the fact that addressing these questions presents great challenges, and this also requires further in-depth investigation of optogenetics. It is worth noting that optogenetic techniques are crucial in terms of both the pathways, mechanisms, and treatment of NP. Identifying pain nociceptors through the spatiotemporality of optogenetic techniques and identifying damaged neurons is a breakthrough step in understanding the mechanism by which God carries out pain, and provides a crucial role for later treatment.

## Conclusion and outlook

Neuropathic pain is a complex, comprehensive disorder that is extremely challenging to treat clinically, owing to the complexities that surface during NP treatment. The treatments of NP are a major challenge for effective clinical practice. Pharmacotherapy is a common clinical method to relieve pain in patients, but we cannot ignore the side effects of the drug treatment, and its efficacy has not achieved the ideal effect. Non-pharmacological treatments have small side effects, but most of their efficacy lacks reliable clinical evidence. The combination of treatment methods may be a good way to improve the efficacy, as the patients stand to benefit from the cumulative effect of both these treatment methods. It has been clinically confirmed that drug treatment with some traditional treatments can not only increase the efficacy, but also reduce the side effects. It can be further explored in the combination of multiple treatment methods.

In summary, there are uncertainties in both pharmacological and non-pharmacological treatments, and although the efficacy of some treatment methods is clinically affirmed, further research is still needed. Optogenetics, as an emerging field of NP, has unlimited potential in terms of the mechanism pathways and treatment of NP. Through the spatiotemporality of optogenetic techniques, the identification and localization of pain receptors, damaged neurons, which helps us to further explore pain transmission pathways. In addition, optogenetic techniques are now widely used in various fields, including the treatment of NP. According to the previous description, optogenetic techniques have been affirmed for their efficacy in relieving pain, but there are still issues that need to be further explored. For example, optogenetics is currently limited to animal experiments, so how can it be applied to human beings? How is the light-sensitive protein entered? The length of optogenetics to be maintained is also a limitation. Is there a way to make the effect longer or even permanent? There is no denying the fact that addressing these questions presents great challenges, and this also requires further investigation of optogenetics.

Generally, before performing clinical experiments on human beings, we usually conduct experiments first on non-human primates that bear closeness to humans both in evolution and in development. The implementation of optogenetic technology is good and successful in rodents, but we face tough challenges while applying this technology to primates, which also signifies the application of optogenetic technology to clinical practice. Second, optogenetic techniques may be used in combination with other fields (such as slice electrophysiology (89)), to investigate the mechanisms of neuropathic pain further. Examples include changes in the network of connections in brain regions related to NP, and pain pathways involved in nerve pain. The combination of optogenetic techniques with other techniques is going to work wonders and is thus likely to help explore the underlying mechanisms of NP even further. At the same time, this technology also has great potential in exploring the potential targets of NP treatment and the development and efficacy of potential drugs. If this technique can identify its damaged pain neurons, it provides a potential therapeutic target and it marks a breakthrough in neuroscience. Optogenetics technology is going to be of immense help to medical fraternity and patients by being able to judge the positioning of drugs after they enter the body through their spatial advantages. This in turn helps to pave the way to explore the mechanism of action of drugs and the possible mechanism of their own diseases, and then to judge the efficacy of their drugs. The application of this technology also reduces the side effects consequent to the use of many clinical drugs, and the above conditions are possible research directions in NP optogenetics in the future and have great potential.

In a word, optogenetics brings with it a reliable promise for the treatment of NP. After analyzing the various advantages of optogenetics for the treatment of NP, we can emphatically state that the therapeutic prospects of optogenetic technology for NP are certain and deserve promotion and exploration for extensive experimental research.

## Author contributions

SL, XF, and HB have participated sufficiently to accept responsibility for the content of the manuscript. SL wrote the draft of the article. XF and HB played a critical role in article revision. All authors contributed to the article and approved the submitted version.

## Funding

This work was supported by the Kunming Medical Joint Project-Key Project (202101AY070001-001), Kunming Medical University 100 Young and Middle-Aged Academic and Technical Backbone Training Program (J13326055), and Yunnan Province Ten Thousand Talents Program Young Top Talent Special Project (YUWR-QNBJ-2019-043).

## Conflict of interest

The authors declare that the research was conducted in the absence of any commercial or financial relationships that could be construed as a potential conflict of interest.

## Publisher's note

All claims expressed in this article are solely those of the authors and do not necessarily represent those of their affiliated organizations, or those of the publisher, the editors and the reviewers. Any product that may be evaluated in this article, or claim that may be made by its manufacturer, is not guaranteed or endorsed by the publisher.
